# Impact of Parenting Styles and Socioeconomic Status on the Mental Health of Children

**DOI:** 10.7759/cureus.43988

**Published:** 2023-08-23

**Authors:** Hemal Makwana, Kiran Kumar Vaghia, Viren Solanki, Vedant Desai, Rithik Maheshwari

**Affiliations:** 1 Psychiatry, Gujarat Medical Education and Research Society (GMERS) Medical College and Hospital, Valsad, IND; 2 Psychiatry, Government Medical College, Baroda, Valsad, IND

**Keywords:** mental health disorders, adolescent mental health, poverty, child mental health, parenting styles

## Abstract

Background

The relationship parents share with their children is unique and very important for their overall growth and development. Parenting is classified into the following four types: authoritarian, authoritative, permissive, and uninvolved. This study aimed to understand the relationship between socioeconomic status and parenting styles adopted by parents and compare various factors affecting the mental health status of children.

Methodology

An observational cross-sectional study was conducted among 480 students from four different schools in Valsad, Gujarat, India. The chief parenting style of both parents was determined, and the Pediatric Symptom Checklist (PSC) scores were calculated for the students. Data were analyzed and various tests of significance were performed.

Results

There was a highly significant association between various parenting styles adopted by both parents and the PSC score of children. Interparental consistency showed a lower score on the PSC scale. There was a moderate positive correlation between an authoritarian parent and the poor mental health status of the child. As age advanced, children were seen to experience more emotional and psychological troubles. The education of the mother had a significant association with the well-being of the child. However, there was no impact of socioeconomic status on parenting style and PSC score.

Conclusions

Poor parenting technique contributes to various psychological problems in children with advancing age. The involvement of healthcare facilities in this field at the earliest will ensure a better environment for the child to grow and learn.

## Introduction

According to Darling and Steinberg, parenting style is defined as a constellation of attitudes and behavior of parents toward their children and the emotional climate in which they express their behaviors [1]. Kaniusonyte and Laursen in their person-oriented longitudinal study described three core behavioral dimensions of parents from an adolescent point of view, namely, behavioral control, psychological control, and support [2]. Behavioral control includes the following two parameters: demandingness and responsiveness. Demandingness or control refers to all impositions that a parent makes on the child to become integrated into the family by their maturity demands, strict supervision, disciplinary efforts, and punishments if the child fails to obey [2]. Responsiveness or acceptance refers to the extent to which parents can nurture individuality and self-regulation by being supportive, kind, and compliant with a child’s special demands [3]. Based on these two parameters, Ballantine classified parenting into the following four styles: authoritarian parenting (high demandingness with low acceptance), authoritative parenting (moderate demandingness and acceptance), permissive parenting (high acceptance with low demandingness), and uninvolved parenting (neither demanding nor accepting) [3]. Psychological control refers to behavior that invades a child’s personal space and manipulates his/her thinking and emotions, whereas support refers to behavior that promotes the emotional as well as psychological well-being of the child. It helps to develop healthy and harmonious parent-child relationships [2]. Gupta and Mehtani developed a scale in 2017 that classified parenting into the following four styles: democratic, autocratic, permissive, and uninvolved, similar to that proposed by Ballantine [4].

Authoritarian parents have high demandingness or control, but they lack responsiveness or acceptance. Parents often fail to understand the emotional needs of their children. There are preset rules and regulations that the children are expected to follow. If the child is unable to meet the expectations, there are repercussions. Maccoby and Martin in their study described that authoritarian parents attach strong values to maintain their authority and suppress all the efforts of children that are made to challenge them [5]. There is very little heart-to-heart communication between both generations. Erikson’s theory of psychosocial development clearly shows the huge impact the early years of life have on the development of personality [6]. The child may grow up having low self-confidence, difficulty expressing, inability to trust, and isolation from the surroundings. The child is conditioned to work hard to gain the parent’s love as a reward. Even as a grown-up, it becomes the primary nature of the person to gain validation from others. In contrast, there even are chances that the child may develop an antisocial personality. They may become rebellious to grab their parent’s attention.

Permissive parents have high responsiveness or acceptance, but they lack demandingness or control. There are very little or no restrictions on the child. They tolerate all their actions and fulfill all their demands. Children are supposed to make decisions for which they might not even be ready. They may have good confidence and social skills, but it can also give them a huge surge of ego. They do not appreciate the things that come to them easily, and it becomes difficult for them to accept the responsibilities that life has to offer later on.

Authoritative parents have high demandingness or control as well as high responsiveness or acceptance. They are strict with rules, but they are also open to understanding and change for their child. It can be considered a healthy way of parenting. Children grow to be happy, responsible, confident, and successful [3]. Even in this study, positive and best results are seen under the influence of authoritative parenting.

Uninvolved parents are neglectful. They lack both demandingness or control and responsiveness or acceptance. They just provide the basic needs of life and get busy in their own lives. Children raised by uninvolved parents are low in self-esteem and are less competent compared to their peers [3]. They feel left out and utterly alone. The prevalence of depression might also be high in such cases.

According to Alfred Adler, the personality of an individual starts developing in early childhood. He believed that lifelong coping strategies adopted by each person depend on how deeply he/she is connected to the family. When we are encouraged, we feel confident and valued. When we are discouraged, we feel depressed and tend to give up [7]. The original classification by Baumarind divided parenting into authoritarian, authoritative, and permissive styles [8]. This was later elaborated by Martin and Maccoby [5].

Parenting varies from generation to generation. Traditional Indian parenting was based on life experiences, cultural and religious values of the community, and gender preferences to some extent. Authoritarian parenting was very common and was highly influenced by how the parents themselves were raised in their childhood [9]. However, studies have shown that with time there has been an increase in the number of permissive parents while authoritative and authoritarian parenting shows a declining trend [10]. Many factors can contribute to this such as modernization, improvement in literacy rate, urbanization, social media, and cultural and religious beliefs.

## Materials and methods

This was a cross-sectional, questionnaire-based, observational study conducted in four different schools in Valsad, Gujarat, India. Participants were selected by the lottery method. Data collection was done in August and September 2022. Approval of the Institutional Human Ethics Committee, Gujarat Medical Education and Research Society Medical College & Civil Hospital, Valsad (approval number: IHEC/39/'22) was taken before starting the study. Written permission to conduct the study was taken from the principals of all the schools. The purpose and procedure of the study were explained to teachers and students in the class by the investigator. Those who refused to participate were not included. Confidentiality was maintained throughout the study.

Study tools

The sociodemographic assessment form included variables such as age, sex, type of family, religion, education and occupation of both parents, and per-capita income.

The Parenting Style Questionnaire is a 30-item structured questionnaire having a six-point Likert scale-type response format. Three parenting styles are assessed under different headings, i.e., authoritarian, authoritative, and permissive. The one with the highest score is considered the chief parenting style of the parent. Both parents filled up the form individually.

The Pediatric Symptom Checklist (PSC) and the Youth-Pediatric Symptom Checklist (Y-PSC) is a 35-item structured questionnaire with a three-point Likert scale-type response format. A high PSC score suggested poor mental health. PSC was given to the age group 7-12 years with a cut-off score of 28. A score higher than the cut-off was concluded as impaired or poor mental health status. Y-PSC was given to the age group 13-18 years with a cut-off score of 30.

Study procedure

A total of 120 students were selected randomly from each school, five girls and five boys from each class starting from class 1 to class 12. Therefore, the sample size was 480. A brief introduction was given in every class of all the schools about the study and the procedure with the permission of the principals of all the schools. Subsequently, a set participant information sheet, informed consent form and assent form, sociodemographic assessment form, parenting style questionnaire, and PSC or Y-PSC was sent home with the participants. They were provided in vernacular languages also. All forms were collected from the school within a week. Contact numbers of investigators were provided for any queries.

Data analysis

All collected data were entered in Microsoft Excel and analyzed using SPSS (IBM Corp., Armonk, NY, USA). Various statistical tests were used to determine significant associations and correlations between different variables.

## Results

The study included 480 participants with a 1:1 boy-to-girl ratio. The mean age of the study participants was 12.5 ± 3.46 years. The mean age of fathers was 41.75 ± 3.21 years, and the mean age of mothers was 37.15 ± 4.18 years. The distribution of sociodemographic variables is presented in Table 1.

**Table 1:** Demographic profile of participants.

Demographic factor	Total (%)
Age	
7–12 years (children)	240 (50%)
13–18 years (adolescents)	240 (50%)
Gender	
Male	240 (50%)
Female	240 (50%)
Religion	
Hindu	435 (90.63%)
Muslim	31 (6.45%)
Christian	14 (2.92%)
Type of family	
Nuclear	320 (66.67%)
Joint	160 (33.33%)
Education of father	
Illiterate	3 (0.63%)
Primary	35 (7.29%)
Secondary	91 (18.95%)
Higher secondary	128 (26.67%)
Intern/diploma	50 (10.42%)
Graduate	114 (23.75%)
Postgraduate	59 (12.29%)
Occupation of the father	
Unemployed	82 (17.08%)
Unskilled worker	13 (2.71%)
Semi-skilled worker	33 (6.88%)
Skilled worker	60 (12.5%)
Clerk and other	144 (30%)
Semi-professional	89 (18.54%)
Professional	59 (12.29%)
Education of the mother	
Illiterate	1 (0.21%)
Primary	19 (3.96%)
Secondary	77 (16.04%)
Higher secondary	130 (27.08%)
Intern/diploma	54 (11.25%)
Graduate	127 (26.46%)
Postgraduate	72 (15%)
Occupation of the mother	
Unemployed/Housewife	369 (76.88%)
Unskilled worker	1 (0.21%)
Semi-skilled worker	5 (1.04%)
Skilled worker	12 (2.5%)
Clerk and other	19 (3.96%)
Semi-professional	64 (13.33%)
Professional	10 (2.08%)
Modified Kuppuswamy socio-economic scale	
Upper	70 (14.58%)
Upper middle	231 (48.13%)
Lower middle	108 (22.5%)
Upper lower	67 (13.96%)
Lower	4 (0.83%)

Of the total participants, the majority were Hindu (90.63%), while 6.45% were Muslim, and the remaining 2.92% were Christian. Overall, 66.67% of children lived in nuclear families and 33.33% lived in a joint family. The average family size was 5.189 ± 1.742. Mothers had received better education compared to fathers. Overall, 41.46% of mothers were graduates, of whom 15% were postgraduates. In comparison, 36.04% of fathers were graduates and 12.29% were postgraduates. Even after good education among women, 76.88% were housewives while the remaining 23.12% had different occupations. Among the men, 82.92% were employed. Most families were middle-class or upper-middle-class, accounting for 48.13% of the population, followed by the lower-middle-class with 22.5%.

A comparison of PSC scores with various variables is shown in Table 2. The overall average PSC score was 12.28. In the age group of 7-12 years, the mean PSC score was 9.64, with only five (2.08%) participants having scores higher than the cut-off value. In the age group of 13-18 years, the mean PSC score was 14.91, with 16 (6.67%) having a higher score. This association between age groups and PSC scores was found to be highly significant (p = 8.26E-13).

**Table 2:** Comparison of the Pediatric Symptom Checklist (PSC) scores and various variables.

Variables	Total, n (%)	Pediatric Symptom Checklist Score, mean (SD)	One-way ANOVA (F)	P-value
Father’s parenting style				
Authoritarian	21 (4.37%)	27.38 (12.50)	43.49	4.46E-18*
Authoritative	421 (87.71%)	11.49 (7.20)		
Permissive	38 (7.92%)	12.63 (8.74)		
Total	480	17.17 (9.48)		
Mother’s parenting style				
Authoritarian	18 (3.75%)	27.11 (11.26)	35.79	3.31E-15*
Authoritative	409 (85.21%)	11.49 (7.51)		
Permissive	53 (11.04%)	13.34 (7.98)		
Total	480	17.31 (8.92)		
Interparental consistency				
Present	394 (82.08%)	11.85 (7.75)	5.915	0.015*
Absent	86 (17.92%)	14.23 (10.17)		
Socioeconomic status				
Upper	70 (14.58%)	13.49 (9.12)	0.774	0.542
Upper middle	231 (48.13%)	12.37 (8.32)		
Lower middle	108 (22.5%)	11.56 (8.16)		
Upper lower	67 (13.96%)	12.04 (7.48)		
Lower	4 (0.83%)	8.75 (6.40)		
Children (7–12 years)	240 (50%)	14.91 (8.56)	54.125	8.26E-13*
Adolescents (13–18 years)	240 (50%)	9.64 (7.06)		
Gender				
Boys	240 (50%)	12.3 (8.40)	0.004	0.952
Girls	240 (50%)	12.25 (8.16)		
Religion				
Hindu	435 (90.63%)	12.30 (8.05)	0.181	0.834
Muslim	31 (6.45%)	12.52 (11.64)		
Christian	14 (2.92%)	11 (6.78)		
Type of family				
Nuclear	320 (66.67%)	12.35 (8.21)	0.074	0.785
Joint	160 (33.33%)	12.13 (8.42)		
Education of the father				
Illiterate	3 (0.63%)	10 (7.81)	0.539	0.779
Primary	35 (7.29%)	12.06 (7.18)		
Secondary	91 (18.95%)	13.52 (7.92)		
Higher secondary	128 (26.67%)	11.77 (8.13)		
Intern/Diploma	50 (10.42%)	11.88 (8.08)		
Graduate	114 (28.75%)	12.46 (9.10)		
Postgraduate	59 (12.29%)	11.69 (8.40)		
Occupation of the father				
Unemployed	82 (17.08%)	12.68 (8.28)	0.917	0.482
Unskilled worker	13 (2.71%)	13.54 (8.28)		
Semi-skilled worker	33 (36.88%)	12.06 (7.54)		
Skilled worker	60 (12.5%)	13.07 (9.39)		
Clerk and other	144 (30%)	11.04 (8.04)		
Semi-professional	89 (18.54%)	12.49 (7.25)		
Professional	59 (12.29%)	13.44 (9.42)		
Education of the mother				
Illiterate	1 (0.21%)	14	2.398	0.027*
Primary	19 (3.96%)	8.89 (7.69)		
Secondary	77 (16.04%)	12.75 (7.83)		
Higher secondary	130 (27.08%)	12.01 (7.88)		
Intern/Diploma	54 (11.25%)	10.89 (7.99)		
Graduate	127 (26.46%)	14.18 (9.59)		
Postgraduate	72 (15%)	10.81 (6.60)		
Occupation of mother				
Unemployed	369 (76.88%)	11.91 (8.26)	0.719	0.634
Unskilled worker	1 (0.21%)	11 (0)		
Semi-skilled worker	5 (1.04%)	16 (15.49)		
Skilled worker	12 (2.5%)	11.83 (9.12)		
Clerk and other	19 (3.96%)	13.37 (6.96)		
Semi-professional	64 (13.33%)	13.75 (8.07)		
Professional	10 (2.08%)	13.3 (7.44)		

Most parents, 87.71% of fathers and 85.21% of mothers, were authoritative. There was a statistically highly significant association between the parenting style of the father and the PSC score (p = 4.46E-18), as well as between the parenting style of the mother and the PSC score (p = 3.31E-15). However, on applying the Z-test, no significant difference was seen between the PSC scores of authoritative fathers and permissive fathers (p = 0.218), as well as between the PSC scores of authoritative mothers and permissive mothers (p = 0.055). Among the parents, 82.08% showed similar parenting styles, while only 17.92% had a different style from each other. This interparental consistency had a statistically significant association with the PSC score (p = 0.015).

Other factors such as socioeconomic status, gender, religion, type of family, education of father, occupation of father, and occupation of mother had no statistically significant association with PSC scores. Education of the mother, however, had a significant relationship with the PSC score (p = 0.027). Table 3 shows that there was no statistical significance between socioeconomic status and parenting style.

**Table 3:** Comparison of parents’ parenting style and socioeconomic status.

	Upper, frequency (%)	Upper middle, frequency (%)	Lower middle, frequency (%)	Upper lower, frequency (%)	Lower, frequency (%)	Total (%)
Father’s parenting style						
Authoritarian	4 (5.72)	11 (4.76)	3 (2.78)	3 (4.48)	0 (0)	21 (4.38)
Authoritative	61 (87.14)	202 (87.45)	95 (87.96)	59 (88.06)	4 (100)	421 (87.71)
Permissive	5 (7.14)	18 (7.79)	10 (9.26)	5 (7.46)	0 (0)	38 (7.91)
Total	70 (100)	231 (100)	108 (100)	67 (100)	4 (100)	480 (100)
P-value, chi-square	0.984, 1.884					
Mother’s parenting style						
Authoritarian	5 (7.14)	8 (3.46)	3 (2.78)	2 (2.99)	0 (0)	18 (3.75)
Authoritative	59 (84.29)	198 (85.72)	88 (81.48)	61 (91.04)	3 (75)	409 (85.21)
Permissive	6 (8.57)	25 (10.82)	17 (15.74)	4 (5.97)	1 (25)	53 (11.04)
Total	70 (100)	231 (100)	108 (100)	67 (100)	4 (100)	480 (100)
P-value, chi-square	0.482, 8.056					

The correlation of PSC scores was compared with various variables, as depicted in Table 4. There was a negative correlation between authoritative parents and PSC scores. Authoritarian parents and age showed a moderate positive correlation while permissive parents showed a mild positive correlation.

**Table 4:** Correlation of the Pediatric Symptom Checklist (PSC) score with various variables.

Variable 1	Variable 2	R
Pediatric Symptom Checklist score	Authoritarian father	0.391
Pediatric Symptom Checklist score	Authoritarian mother	0.354
Pediatric Symptom Checklist score	Authoritative father	-0.254
Pediatric Symptom Checklist score	Authoritative mother	-0.229
Pediatric Symptom Checklist score	Permissive father	0.013
Pediatric Symptom Checklist score	Permissive mother	0.045
Pediatric Symptom Checklist score	Age	0.332

## Discussion

The present study aimed to understand how different factors affect parenting and the mental health status of the child. Of all participants, only 21 (4.375%) were concluded to have impaired mental health status. The parenting questionnaire by Robinson et al. [11] used in this study has been validated before in different studies by Kimble [12] and Önder and Gülay [13]. PSC and Y-PSC scales used have also been used before by Navon et al. [14].

There was a highly significant association between age and mental health status of children showing some troubles and an increase in stress with aging in children. Kuppens and Ceulemans conducted a study involving 600 families raising children aged 8-10 years to gain a better understanding of parenting. They described that the change in the nature of children with age is linked to the physical and cognitive development of the child along with the demands and expectations of society [15].

No significant association was found between gender and the mental health of the child, which is similar to previous studies by Yadav et al. [16] and Sharma and Pandey [17]. The sex of the parent or child was not related to the type of parenting adopted [3]. In a cross-sectional study by Vijay et al. in South India on adolescents, it was noted that the majority of boys were raised by permissive parents and had moderate stress levels whereas the majority of girls were raised by authoritative parents and had low stress levels [18].

Overall, 66.67% of families were nuclear, and it had no significant relationship with a child’s mental health. According to Sondhi, the smaller family size allows better parental indulgence as they can spend more time, energy, and resources on their child [9]. Larger families were common in the Muslim religion. However, there was no significant relationship between religion and the mental health of a child.

The occupation and education of parents are two important parameters in parenting. According to a study by Kalil on the role of parenting in the intergenerational transmission of poverty, educated parents talk more to their children, are more involved, are less punitive, and are more understanding, whereas poor cognitive development was seen in low-income children with less educated parents due to lack of maternal responsiveness [19]. Boediman and Desnawati stated that parents with a bachelor’s degree or higher tended to be authoritative compared to less educated parents [20]. Mothers are called caring figures while fathers are the authority figures. Children usually feel distant from their fathers as most are providers in the family and spend less time at home [15]. However, this study showed a significant association only between the education of the mother and the mental health of the child. Despite the very high literacy rate of mothers (99.79%) with 41.46% of mothers having a bachelor’s degree or higher, only 23.12% were working. More stress is seen among working mothers which affects their relationships with their children. Non-working mothers can provide more emotional and social support [21].

The questionnaire used in this study for parents was designed to assess the three parenting styles, i.e., authoritarian, authoritative, and Permissive. Of the total parents who participated, the majority were authoritative (86.46%), followed by permissive (9.48%), and authoritarian (4.06%). These results were consistent with many previous studies [17,20,22,23]. This study showed a statistically significant association between the parenting style of both fathers and mothers and the mental health of their children. With changing times, it has been proven that parenting affects children in many ways. A study by Yadav et al. reported that authoritative parenting is most suitable for better emotional intelligence in children to handle complex situations in life [16]. Sharma and Pandey showed that adolescents with authoritative and permissive parents had higher levels of self-confidence [17]. Caring character, better school engagement, and higher self-esteem were related to authoritative parents [2]. Seth and Ghormode found a positive and significant association between authoritative parenting and academic achievement in high school [24], whereas Gupta and Mehtani proved the same in senior secondary school students [4]. One study concluded that authoritarian parents considered their children difficult to handle [23]. High levels of surgency and more aggressive behavior were common in children with authoritarian fathers [25]. Aggressive and hostile behavior toward peers is a result of emotional and behavioral maladjustment [26]. Such aggressive and antisocial behavior can be controlled by greater affection, communication, and humor [27]. Thus, parenting affects a child’s behavior, but the directionality remains unknown [28].

Sriram considered poverty and common mental disorders as inseparable twins [29]. Inadequate housing, poor education, malnutrition, large families, and insecurity contribute to poverty, which, in turn, affects the mental health of an individual [30]. Cooper also showed that mothers of lower economic status had poor parenting skills. The present study does not conclude the same. Socioeconomic status had no significant relationship with the mental health of the child or parenting. This was consistent with the study by Joseph and John [3].

## Conclusions

This study aimed to explain how various factors affect parenting and, in turn, affect the mental health status of the child. A highly significant association was seen between the parenting style of both parents individually with the mental health of their child. Authoritative parenting was considered the best whereas authoritarian parenting showed a positive correlation with poor mental health. Interparental consistency also had a significant relationship with the child. An impaired psychological condition was noted in older children. No statistically significant relationships were seen with gender, type of family, religion, occupation and education of the father, occupation of the mother, and socioeconomic status. However, the education of the mother had an impact on the child’s well-being.

It is recommended to conduct a longitudinal study in the future to better understand these relationships. A larger and more diverse population can also be recruited to include social and cultural aspects as well. This study can be used in the future to design and organize various parenting programs for parents to improve their skills and develop harmonious and healthy relationships with their children. Integrated sessions with parents along with their children can be encouraged. Healthcare facility involvement in the tender early age of the child can prevent the development of common mental health disorders in the later stages of life. With changing times, it is necessary to understand and adopt the modern mindsets of the new generation, but it is equally important for parents to teach them morals and values. This certainly requires effort on both ends. More studies are encouraged in this field to overcome the problems we face today.
